# Lab-on-a-brain: Implantable micro-optical fluidic devices for neural cell analysis *in vivo*

**DOI:** 10.1038/srep06721

**Published:** 2014-10-22

**Authors:** Hiroaki Takehara, Akira Nagaoka, Jun Noguchi, Takanori Akagi, Haruo Kasai, Takanori Ichiki

**Affiliations:** 1 Department of Bioengineering, School of Engineering, The University of Tokyo, 2-11-16, Yayoi, Bunkyo-ku, Tokyo. 113-8656, Japan; 2 Laboratory of Structural Physiology, Graduate School of Medicine, The University of Tokyo, 7-3-1, Hongo, Bunkyo-ku, Tokyo. 113-0033, Japan

## Abstract

The high-resolution imaging of neural cells *in vivo* has brought about great progress in neuroscience research. Here, we report a novel experimental platform, where the intact brain of a living mouse can be studied with the aid of a surgically implanted micro-optical fluidic device; acting as an interface between neurons and the outer world. The newly developed device provides the functions required for the long-term and high-resolution observation of the fine structures of neurons by two-photon laser scanning microscopy and the microfluidic delivery of chemicals or drugs directly into the brain. A proof-of-concept experiment of single-synapse stimulation by two-photon uncaging of caged glutamate and observation of dendritic spine shrinkage over subsequent days demonstrated a promising use for the present technology.

The availability of an experimental technique that can be used to investigate cell-cell communication in intact neural networks has provided a powerful key for understanding more about the brain. Recent advances in two-photon laser scanning microscopy (2PLSM) have enabled neural cells inside thick brain tissues to be observed with sub-cellular resolution^1^,^2^,^3^,^4^. An optical microscopic approach has several advantages over an electrophysiological approach, for example, 1) it is relatively less invasive, 2) spatial and temporal information can be simultaneously acquired, 3) neurons can be identified using a genetic approach, 4) optical techniques such as optogenetics or the uncaging of caged compounds can be applied.

Thus the analysis of neurons in the brain of a living animal by 2PLSM has been explored in an attempt to understand brain functions including higher functions such as learning and memory^5^,^6^. However, the *in vivo* imaging of neurons in living animals is not straightforward since the opacity of the skull bone usually prevents optical imaging. Even when the skull bone is surgically removed, the loss of brain fluids and bacterial infection at the exposed brain tissues are often critical problems. Therefore, in the last decade, extensive effort has been devoted to overcoming these technical issues, and two surgical methods have been developed. One is the cranial-window method involving replacing the bone with a cover glass^7^,^8^,^9^,^10^ and the other is the thinned-skull method involving thinning the bone to the order of 10 μm^11^,^12^,^13^. Both methods can provide an optical path for microscopic imaging, prevent external infection, and preserve the physiological conditions of the brain tissue. In particular, the thinned-skull method, which does not involve surgical dissection of skull bone, is less invasive.

Moreover, the combined use of the 2PSLM and photoresponsive compounds has been shown as an advanced approach for obtaining valuable insights into the dynamic activities of neurons^14^,^15^,^16^. Two-photon uncaging enables highly localized synapse stimulation, even at a single-spine level, via the photolytic release of caged neurotransmitters^17^,^18^,^19^. Noguchi *et al.* reported an extension of the two-photon uncaging in the study of neocortical pyramidal neurons of adult mice *in vivo*^19^. In their work, the chemical compounds were applied to the neocortex by partially covering the skull opening with a glass coverslip and by attaching a ring-shaped open chamber on the skull as a drug inlet reservoir. This method which is a modified version of the cranial window method was useful for *in vivo* two-photon uncaging, but can only be used for single-day experiment due to the inevitable risk of bacterial infection of the exposed brain.

In this paper, we propose a new experimental platform, called a “lab-on-a-brain”, formed by replacing a small part of the skull bone of a mouse with an implantable micro-optical fluidic device that enables both the long-term imaging of neurons *in vivo* and the delivery of chemicals into brain tissues. The drug delivery method mainly used in neuroscience is the cannula method, which is relatively invasive and sometimes obstructs microscopic observation. Thus, we attempted to achieve both optical observation and the delivery of various reagents with low tissue damage. The design, the fabrication process including sterilization, and the method of installation of the implantable device are described in detail. To demonstrate the potential use of the implantable device, the time course of the formation and elimination of spines on the same dendrite was tracked in combination with 2PLSM for more than seven weeks. We also observed the spine shrinkage *in vivo* after two-photon uncaging stimulation for the first time. The spine shrinkage is considered as a structural correlate of the long-term depression of excitatory spine synapses^20^.

## Results

### Design and fabrication of the micro-optical fluidic device

**Figure 1:**
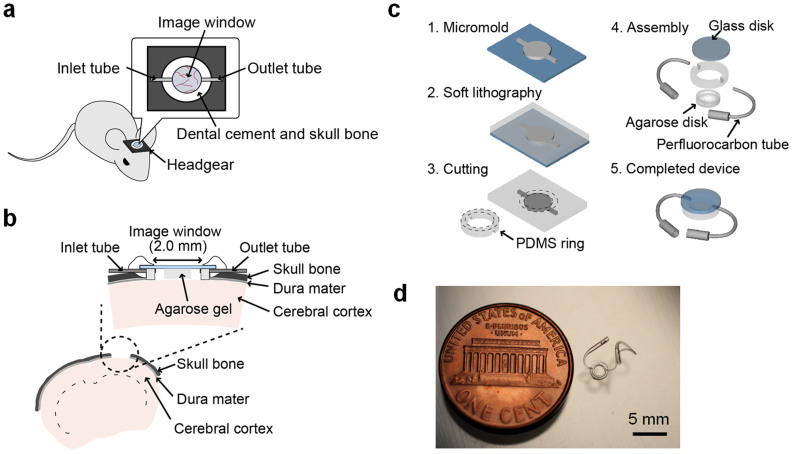
Design and fabrication process of the implantable micro-optical fluidic device. (a) Schematic of a mouse implanted with the device. The exposed cortex can be observed through the device window. Chemicals can be introduced into the device via an inlet tube and removed via an outlet tube. (b) Schematic cross section of the mouse brain tissue and the implantable micro-optical fluidic device mounted on a mouse skull. Neurons in layers I–VI of the cerebral cortex of a mouse can be observed by 2PLSM. (c) Fabrication process of the implantable micro-optical fluidic device. (d) Photograph of the implantable microfluidic device. The main body of the device is 2.7 mm in diameter and 450 μm in thickness, to which two tubes for the inlet and outlet are connected. Scale bar, 5 mm. The schematic illustrations were drawn by H. T. using Adobe Illustrator software (CS6, Adobe Systems Inc., San Jose, CA, USA) and the photograph was taken by H. T.

Figures 1a and b show schematics of the implantable micro-optical fluidic device developed in this study. The device was designed to be implanted into a certain part of the skull bone to fill a drilled hole. The diameter of the imaging window was chosen to be 2.0 mm, which allows a focused and collected light beam to pass via a high-numerical-aperture (NA) objective lens of the 2PLSM system without any shielding or distortion of microscope images. To achieve high-resolution *in vivo* imaging, a small agarose gel disk was inserted in the gap between the imaging window and the brain surface to prevent pulsatile motion of the imaging site, which is caused by changes in blood pressure and by respiratory motion. Chemicals can be injected into the device via perfluorocarbon tubes and transported into the brain tissue by diffusion. Figure 1c shows the fabrication process of the implantable micro-optical fluidic device. As seen in the fourth assembly step, the device is mainly composed of three elements: a top glass disk, a bottom poly(dimethylsiloxane) (PDMS) part having microstructures patterned by soft lithography, and connection tubes for the chemical inlet and outlet. In addition, the device fabrication is followed by a sterilization step to prevent infection as described in the Methods section. Figure 1d shows the completed device. The main body was 2.7 mm in diameter and 450 μm in thickness. The device can deliver chemicals into the brain tissue at a rate of 10 μLmin^−1^ for 20 min (Supplementary Fig. S1a). In combination with numerical simulation, we can quantitatively predict and control the chemical concentration delivered into the imaged tissue region (Supplementary Figs. S1b and c, Supplementary Movie 1).

### Device implantation on a mouse brain

**Figure 2:**
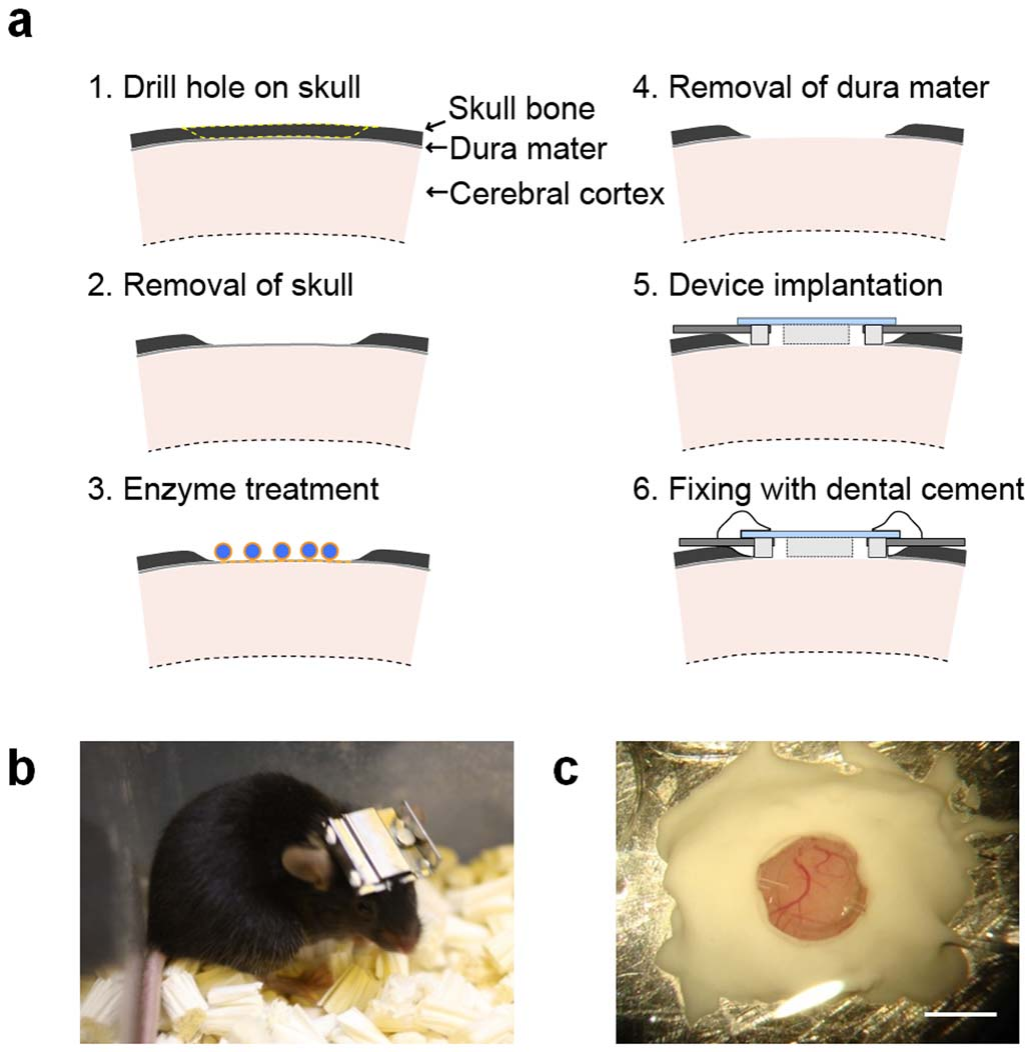
Device implantation procedure and a mouse implanted with the device. (a) Low invasive device implantation procedure with dura removal using enzymatic treatment. (b) Photograph of a living mouse with the headgear attached on its head. The micro-optical fluidic device was protected by placing it inside metal headgear with a detachable cover. (c) Close-up photograph of the implanted device after fixing it mechanically with dental cement. Scale bar, 2 mm. The schematic illustration was drawn by H. T. using Adobe Illustrator software and the photographs were taken by A. N.

The procedure of device implantation into the skull bone of a mouse is depicted in Fig. 2a. We named this low invasive surgery procedure “the systematic skull-dura removal (SSDR) method”, whose details are described in the Methods section. After device implantation, headgear for immobilizing a mouse head on a microscope stage was attached to the mouse, and the mouse was housed in a standard cage, as shown in Fig. 2b. In the present study, the device was implanted over the primary visual cortex. The brain surface can be clearly observed through the imaging window of the device as shown in Fig. 2c.

From the assessment of expression levels of markers of injury/inflammation^21^, we confirmed that no recognizable damage or inflammation was induced in the brain of the mouse that underwent SSDR surgery (Supplementary Fig. S2).

### Long-term observation of neurons in living mice by 2PLSM

**Figure 3:**
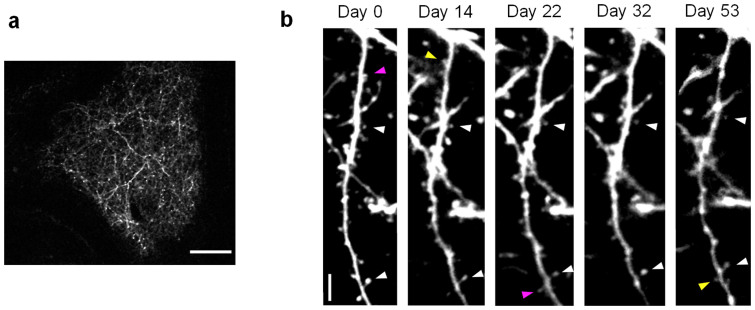
Long-term 2PLSM images of mouse primary visual cortex of a thy1 YFP-H line mouse implanted with the device. (a) A low magnification image. Scale bar, 50 μm. (b) Time-lapse images of a dendrites of the cortex taken over 53 days. The same dendrite of a neuron was observed from the cortex of the mouse implanted with the device. Note that there are stable, newly formed, and eliminated spines (indicated by white, yellow, and magenta arrowheads, respectively). Scale bar, 5 μm. Representative data from 5 experiments.

Figure 3a shows a microscopy image of the brain tissue observed through the window of the implanted device. A high-resolution (<1 μm) microscopy image of neurons was successfully obtained by 2PLSM (see also Supplementary Movie 2). A spacer material with a suitable stiffness is important to suppress the motion of the brain surface without inducing the undesirable physical stimulation of inflammatory cells. Note that the high solute permeability of the gel is also a key property in delivering chemicals uniformly onto the brain surface. As a result, as shown in Fig. 3b, we could achieve long-term observation of the same neurons in the mouse brain by 2PLSM at intervals of several days for 53 days after the surgery. When bleeding from the dura mater and the inflammation of the brain were avoided, hardly any clouding on the imaging window was observed^10^. During the observation, no unnatural changes were observed in the shape of the dendrite. The turnover of synapse formation and elimination was comparable to that reported in a study using the thinned-skull method^13^, indicating the low invasiveness of the present method.

### Single-synapse stimulation by 2PUM

**Figure 4:**
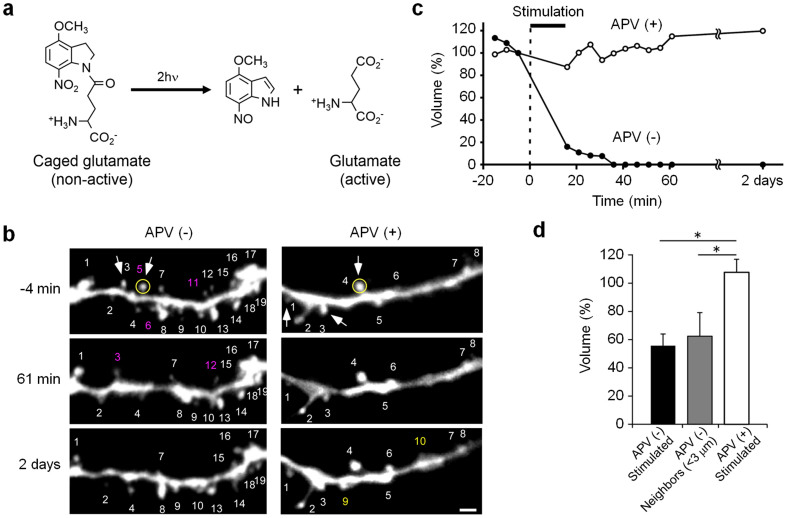
Synapse stimulation by two-photon uncaging microscopy (2PUM). (a) Reaction of photolytic release of MNI-glutamate. (b) 2PSLM photographs of the dendrite of a mouse before stimulation (−4 min) and 61 min and 2 days after uncaging. The photosets on the left- and right-hand sides show the results of APV(−) and APV(+) cases, respectively. Arrows indicate spine synapses stimulated by photo-uncaging. Stable, newly formed, and eliminated spines are indicated by white, yellow, and magenta numbers, respectively. Scale bar, 2 μm. (c) Time course of spine-head volume of the stimulated synapse. The plotted values correspond to the spines indicated by yellow circles in Fig. 4b. Stimulated spine synapses (APV(−)) showed apparent shrinkage within 1 h and their termination was confirmed by the observation 2 days after the stimulation. In contrast, a synapse stimulated in the presence of receptor antagonist (APV) did not show any shrinkage. (d) Average volume of spines subjected to the LTD protocol (17 spines, 4 dendrites, 4 mice), their neighbors within 3 μm (7 spines, 4 dendrites, 4 mice), and the spines subjected to the LTD protocol in the presence of APV (6 spines, 2 dendrites, 2 mice) evaluated 2 days after the stimulation experiment by photo-uncaging. Error bars, s.e.m.; *P < 0.05 by Steel's test; APV (−) stimulated and APV (−) neighbors versus APV(+) stimulated.

The implantable micro-optical fluidic device was applied to two-photon uncaging microscopy (2PUM) accompanied by the acute delivery of chemicals (Fig. 4a). As shown in Fig. 4b, two spine synapses on the same dendrite in the mouse brain were stimulated by uncaging MNI-glutamate at 2 Hz for 15 min, namely, the long-term depression (LTD) protocol was followed^17^,^19^. Figure 4b shows typical 2PSLM photographs of the dendrites before and after stimulation by glutamate uncaging. The changes in the spine head volume are plotted in Fig. 4c. The shrinkage of stimulated spine synapses occurred within 60 min and the elimination of the spines continued for two days after stimulation (Fig. 4b, spines indicated by arrows in the photoset of APV(−) and Fig. 4d). Importantly, we found that the shrinkage was induced even in the spines neighboring to stimulated ones within 60 min and two days later (Fig. 4d), as it was reported for brain slice preparations *in vitro*^20^,^22^.

In contrast, no shrinkage of spine synapses was observed in the presence of NMDA receptor antagonist APV, which was used in a control experiment to verify the phototoxicity of laser irradiation (Fig. 4b, spines indicated with arrows in the photoset of APV (+) and Fig. 4d). Thus, the shrinkage of spines by photo-uncaging stimulation has been observed *in vivo* over 2 days for the first time. It is noteworthy that, unlike our device approach, conventional open-skull surgeries do not allow chronic focal application of chemicals, and cannot study long-term effects.

## Discussion

Microfluidic devices have already shown great potential for biological and medical studies *in vitro*^23^,^24^,^25^,^26^,^27^,^28^. The present study aimed to further extend the potential use of microfluidic technology to provide a revolutionary interface device for *in vivo* cellular research. The highest priority issue of the implant device is to minimize pain and distress to animals used for research. We need to pay careful attention not to induce immunological rejection due to the device implantation. The original purpose of *in vivo* imaging cannot be achieved when an immunological response disturbs the physiological state of cells under observation. In this study, we specifically developed our device for experimental studies using living mice. There are many reasons for using experimental mice in addition to their small size and ease of breeding^29^. The small size of the mouse brain makes it suitable for *in vivo* imaging of neurons since whole layers of the neocortex can be observed using 2PLSM. Moreover, various genetically engineered mice are now available for biological and medical research^30^,^31^,^32^,^33^,^34^. To reduce the pain and distress to mice, a technical challenge is to make the device as small as possible. In the previous literature, a pial window crown of 10 mm diameter for use in rats was reported^35^, and a glass window of 32 mm diameter was also reported for use in monkeys^36^. In contrast, the cranial window sizes for mice are limited to 2–4 mm in diameter^10^. Hence the introduction of microdevice technology is necessary to fulfill the requirements for downsizing without degrading functionality for optical observation and chemical delivery.

Next, we discuss the impact of long-term observation of the mouse brain *in vivo*. So far, there has been no means of performing long-term observation of a neuron or dendrite of a portion controlling the brain environment using a reagent since inserting cannula causes considerable injury of the tissue. Recently, controlling the flow of cerebrospinal fluid has been found to be important for the normal activity of the central nervous system, and its failure may be a cause of various diseases including Alzheimer's disease^37^. Using our method, the brain environment can be readily modified with less brain damage.

Moreover, higher brain functions such as learning and memory are phenomena involving relatively long periods of time ranging from several days to a few months. Also, the long-term sequential analysis of neural cells and biochemical molecules is required to clarify the mechanisms and to develop therapeutic methods for intractable brain diseases such as Alzheimer's disease, Parkinson's disease, and Fragile X syndrome^38^,^39^. Our implantable micro-optical fluidic device is expected to open a door to such less-explored but important biological and medical research targets.

In summary, an implantable micro-optical fluidic device was developed, which enables both the long-term microscopic observation of neurons in a mouse brain and the delivery of chemicals into the brain tissue. The surgical scheme that was systematically developed for low invasive installation of the device enabled the physiological condition of the intact brain to be essentially preserved. Consequently, the long-term tracking of the same dendritic spines by 2PLSM over a period of weeks has become possible. Moreover, a proof-of-concept experiment involving 2PUM for single-synapse stimulation in a living brain was conducted as an example of an application of the present technique. The experimental “lab-on-a-brain” platform using an implantable micro-optical fluidic device is expected to contribute to the advancement of neuroscience and neurological medicine.

## Methods

All experimental protocols were approved by the Animal Experiment Committee of School of Medicine, the University of Tokyo and all care of animals and experimental procedures were carried out in accordance with the Guidelines for the Care and Use of Laboratory Animals of the Department of Medicine, the University of Tokyo.

### Device fabrication

The top glass disk of the device was fused silica glass (Sendai Sekiei Ltd., Sendai, Japan) of 2.7 mm diameter and 150 μm thickness. The bottom PDMS part was prepared by soft lithography^40^. To form a microchannel mold, a 225-μm-thick dry film photoresist (TMMF 2045, Tokyo Ohka Kogyo Co. Ltd., Tokyo, Japan) was laminated onto a glass substrate, exposed to UV light through a chrome photomask, and developed. A PDMS prepolymer (Sylgard 184, Dow Corning Co., MI, USA) was cured at 85°C for 120 min on the microchannel mold and the slab of PDMS of 315 μm thickness was peeled off from the mold. Then, the PDMS sheet including microchannels of 200 μm width and 225 μm height was cut into a ring shape of 2.0 mm inner diameter and 2.7 mm outer diameter. Then, the PDMS ring and the top glass disk were bonded together by heating at 235°C for 150 min. Finally, two perfluorocarbon tubes of 200 μm outer diameter were connected to the PDMS ring to form the inlet and outlet of chemicals. After completing the fabrication process, the device was placed in a sterilization pouch (Fisher Scientific, PA, USA) and sterilized in a steam autoclave operated on a liquid cycle at 2 atm, 121°C for 20 min.

### Surgical implantation

Implantation experiments using the SSDR method were performed as follows. The surgical procedure was performed carefully to avoid inflammatory reaction of the brain tissue. An adult mouse expressing GFP or YFP in a subset of neurons (Thy1 GFP-M or YFP-H line, >8 weeks old) was initially anesthetized by injecting a combination of ketamine and xylazine (Rompun, Bayer Health Care, Leverkusen, Germany). The headgear used for immobilizing a mouse on a microscope was attached to the mouse head. A 2.7-mm-diameter hole was carefully drilled into the skull exactly over the primary visual cortex (3.0 mm posterior, 2.5 mm lateral to bregma) using a dental drill. After the removal of the skull bone, the exposed dura mater was washed with artificial cerebrospinal fluid (ACSF) (125 mM NaCl, 2.5 mM KCl, 1.25 mM NaH_2_PO_4_, 26 mM NaHCO_3_, 20 mM glucose, 2 mM CaCl_2_, and 1 mM MgCl_2_, sterilized by filtration) including antibiotics (2 mg/mL chloramphenicol, 2500 U/mL polymyxin B sulfate, and 0.25 mg/mL dexamethasone in ACSF)^36^. Then, the dura mater was treated with collagenase (Wako Pure Chemical Industries, Ltd., Osaka, Japan) that was immobilized on Sepharose beads (CNBr-activated sepharose 4B, GE Healthcare, U.K.). This enzymatic treatment facilitates the removal of the dura mater without damaging blood vessels, which can be digested by the enzyme. An agarose gel disk of 1.2 mm diameter and 300 μm thickness, which was sterilized by filtration during preparation, was placed under the device to suppress the pulsatile motion of the brain surface. Finally, the device filled with ACSF was placed in the skull hole and fixed using a resin-modified glass–ionomer cement (Fuji Lute BC, GC Corporation, Tokyo, Japan). After the operation, the mouse was housed in a standard cage with an auxiliary metal cover on the headgear to protect the implanted device.

### *In vivo* two-photon imaging

The mouse was anesthetized with ketamine and xylazine, immobilized under the objective lens of a microscope while wearing the headgear and brain stereotaxis apparatus (SR-5M, Narishige Co., Ltd., Tokyo, Japan), and warmed to 37°C using a heating pad (FST-HPS, Fine Science Tools Inc., North Vancouver, Canada). *In vivo* imaging was performed using an upright microscope (BX61WI, FV1000, Olympus Co., Tokyo, Japan) equipped with Ti-sapphire lasers (Mai-Tai, Spectra Physics Inc., CA, USA). The laser was set at 950 nm for imaging YFP. A water-immersion objective lens (LUMPlanFI/IR NA 0.9 60×, Olympus) was used to observe neurons in the brain.

### *In vivo* two-photon uncaging microscopy

A GFP-M line mouse with the device installed was anesthetized and immobilized onto a microscope. 20 mM of 4-methoxy-7-nitroindolinyl (MNI)-glutamate^41^ was flowed into the channel at a rate of 10 μL min^−1^ using a syringe pump and delivered into the brain tissue for ~30 min before uncaging, and washed off with ACSF at a rate of 10 μL min^−1^ for ~10 min after the imaging session. Single spine synapses were stimulated by uncaging MNI-glutamate using a 720-nm-wavelength pulse laser. Repeated stimulation was performed by 2 Hz for 15 min, namely, the long-term depression (LTD) protocol was followed^17^,^19^. As a control, the stimulation was also performed in the presence of 50 μM D-2-amino-5-phosphonovaleric acid (D-APV), which is the N-methyl-D-aspartic acid (NMDA) receptor antagonist. Spine head volumes were estimated from the total fluorescence intensity by summing the fluorescence values of stacked images of three-dimensional data, as previously reported^17^,^19^.

## Author Contributions

H.T. designed and conducted experiments, analyzed data and wrote the paper. A.N. designed and conducted experiments and analyzed data. J.N. and T.A. contributed to discussions. H.K. and T.I. contributed to discussions and supervised the project. All authors contributed to the editing of the paper.

## Supplementary Material

Supplementary Information Supplementary information

Supplementary Information Supplementary movie 1

Supplementary Information Supplementary movie 2
