# Supplementary material for: Lab-on-a-brain: Implantable micro-optical fluidic devices for neural cell analysis in vivo
Source: Sci Rep. 2014 Oct 22;4:6721. doi: 10.1038/srep06721 (PMC4205880; doi:10.1038/srep06721)
Supplement: Supplementary Information [file srep06721-s1.pdf]

## Supplementary information

# Lab-on-a-brain: Implantable micro-optical fluidic devices for neural cell analysis *in vivo*

Hiroaki Takehara[1]*, Akira Nagaoka[2]*, Jun Noguchi[2], Takanori Akagi[1], Haruo Kasai[2] and Takanori Ichiki[1]

*These authors contributed equally to this work.

Correspondence and requests for materials should be addressed to T. I. (ichiki@bioeng.t.u-tokyo.ac.jp)

[1]*Department of Bioengineering, School of Engineering, The University of Tokyo, 2-11-16, Yayoi, Bunkyo-ku, Tokyo, 113-8656, Japan*

[2]*Laboratory of Structural Physiology, Graduate School of Medicine, The University of Tokyo, 7-3-1, Hongo, Bunkyo-ku, Tokyo, 113-0033, Japan*

## S1. Model study of acute delivery of chemicals into the brain tissue

### S1-1. Experimental Measurement

For the acute delivery of chemicals, the tubes of the device were connected to a syringe. A fluorescent dye solution of 50 μM Alexa Fluor 488 (Molecular Probes Inc., OR, USA) in ACSF was made to flow into the channel of the device at a rate of 10 μL $min^{-1}$ using a syringe pump (BAS Bee pump, Bioanalytical Systems, Inc., IN, USA). The model chemical, the fluorescent dye, was transported from the device into the brain tissue. Time-lapse XZ images of the device and the brain tissue were taken every 10 s for 20 min by 2PLSM. To compare the experimental results with the numerical results, the attenuation of the fluorescence intensity by light scattering in the brain tissue must be considered. According to previous studies[1], the fluorescence intensity decreases exponentially with increasing depth in tissues, $F \propto [P_0 \cdot \exp(-z/l_s)]^2$. Here, $F$ is the intensity of fluorescence generated in the focal plane, $P_0$ is the power incident on the sample surface, $z$ is the imaging depth and $l_s$ is the scattering length in the tissue. $l_s$ was experimentally obtained as an attenuation coefficient by fitting with the above equation.

### S1-2. Numerical simulation

A numerical simulation was performed using a commercially available software package (CFD-ACE+, ESI Group, Paris, France) to evaluate the fluid flow and molecular diffusion in the device and the brain tissue. A solution with a constant concentration (Alexa488, 50 μM) was supplied from the inlet at a rate of 10 μL $min^{-1}$. The free diffusion coefficient of Alexa488 ($D_{Alexa488}=3.0\times10^{-10}$ $m^2$ $s^{-1}$)[2] was used for the fluid and the agarose, while the effective diffusion coefficient of Alexa488 ($D_{Alexa488}$*) in the mouse cortex was estimated by fitting with experimental data.

## S1-3. Comparison between experimental measurement and numerical simulation

The acute delivery of chemicals using the device results in a concentration gradient in the brain tissue because of the restricted mass transfer by diffusion within the extra cellular space. Therefore, to determine the spatial and temporal changes in concentration, the transient diffusion of fluorescent dyes in the brain tissue was investigated experimentally and numerically. Since the drugs that were used in this study, e.g. MNI-glutamate, are small hydrophilic compounds that have similar molecular weight, they are expected to show comparable distribution with Alexa Fluor 488. Supplementary Figure S1a shows typical fluorescence images of a cross section of the brain tissue before the introduction of fluorescein molecules and 20 min after their introduction. Supplementary Figure S1b shows the concentration distribution of dyes calculated by numerical simulation under the corresponding conditions. Fluorescence intensities measured at various depths in the brain tissue (50, 100, 150 and 200 μm) are plotted against time in Supplementary Fig. S1c using symbols. The fluorescence intensity in the brain tissue rapidly increased immediately after the introduction of the dye solution into the device and then tended to gradually increase after 10 min. The numerically predicted concentration distribution was converted into the fluorescence intensity and plotted using solid lines in the same figure. The attenuation coefficient used in this conversion was experimentally obtained as the length constant at the mouse cortex $l_s$=187.7±1.7 μm at 950 nm (mean±s.d., $n$=5). This value is consistent with reported data ($l_s$=~200 μm at 800 nm, rat cortex)[1]. Comparing the results of the numerical simulation and *in vivo* experiments, the effective diffusion coefficient of Alexa488 in the mouse cortex was obtained as $D_{Alexa488}$*=~1.0 ×$10^{-10}$ $m^2/s$, which is consistent with the value calculated using restricted diffusion (RD) theory[3] ($D_{Alexa488}$*＝0.9-1.2×$10^{-10}$ $m^2/s$, rat cortex). In

principle, the good reproducibility of the numerical simulation indicates that the chemical concentration in the device and the brain tissue can be predicted and quantitatively controlled.

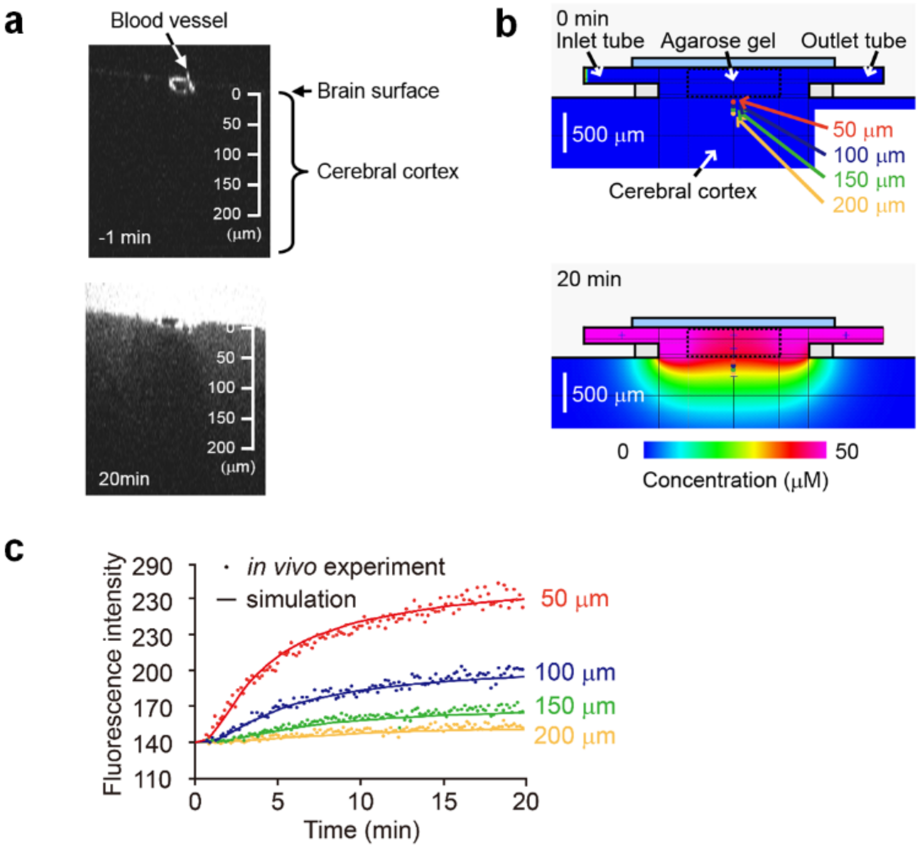


**Supplementary Figure S1** Spatial and temporal changes in concentration with mass transfer in the device and the brain tissue. (a) Fluorescence images of cross section of microchannel before delivery of 50 µM solution of Alexa488 and after delivery for 20 min. (b) Concentration distribution of Alexa488 before delivery and after delivery for 20 min calculated by simulation. (c) Temporal changes in fluorescence intensity during the delivery. Experimentally measured fluorescence intensity (symbols) and numerically simulated fluorescence intensity (solid lines) at various depths in the brain tissue (50, 100, 150 and 200 µm are shown).

## S2. Assessment of injury/inflammation reaction potentially induced by the device implantation

### S2-1. Methods

As a positive control, an implantation procedure with mechanical stimulation to the brain tissues was performed. As a negative control, the brain tissues of mice without surgery were used. An assessment of the number and morphology of microglia using anti-Iba1 immunostaining was conducted in accordance with the method reported in the literature[4]. Anti-IBA-1 antibody (019-19741, Wako Pure Chemical Industries, Ltd., Osaka, Japan), blocking buffer containing 2% horse serum, 0.01% Tween20, 0.1% $NaN_3$ in PBS and Alexa Fluor 488 goat anti-rabbit IgG (H+L) (A11034, Gibco, Invitrogen Co., NY, USA) were used in the immunostaining experiments. The numbers of microglia in brain tissues from the surface to a depth of 200 μm directly under the device and in the contralateral control side were counted. Then, the ratio of the number of activated microglia in the surgery side to that in the contralateral control side was obtained.

### S2-2. Results

It has been reported that the inflammatory reaction affects the activities of neural cells and synapse dynamics[5]; thus, the avoidance of the injury/inflammation reaction of the brain tissue is essential. To confirm that brains that underwent device implantation are essentially comparable to native brains, we assessed the expression levels of markers of injury/inflammation. Supplementary Figures S2a and b show the results of the assessment of the expression levels of markers of injury/inflammation as the number and morphology of microglia. An increase in the number of activated microglia (291±32 cells $mm^{-2}$, microglia ratio=2.2±0.2, mean±s.e.m., $n$=5) was typically observed in the positive control,

but the number of activated microglia after the low invasive device implantation procedure was 176±23 cells $mm^{-2}$ with a microglia ratio of 1.1±0.02 (mean±s.e.m., *n*=5), which remained at the level of negative control (165±10 cells $mm^{-2}$, the microglia ratio=1.0±0.04, mean±s.e.m., *n*=5) as shown in Supplementary Fig. S2c. Thus, the brain that underwent surgery was essentially comparable to a native brain.

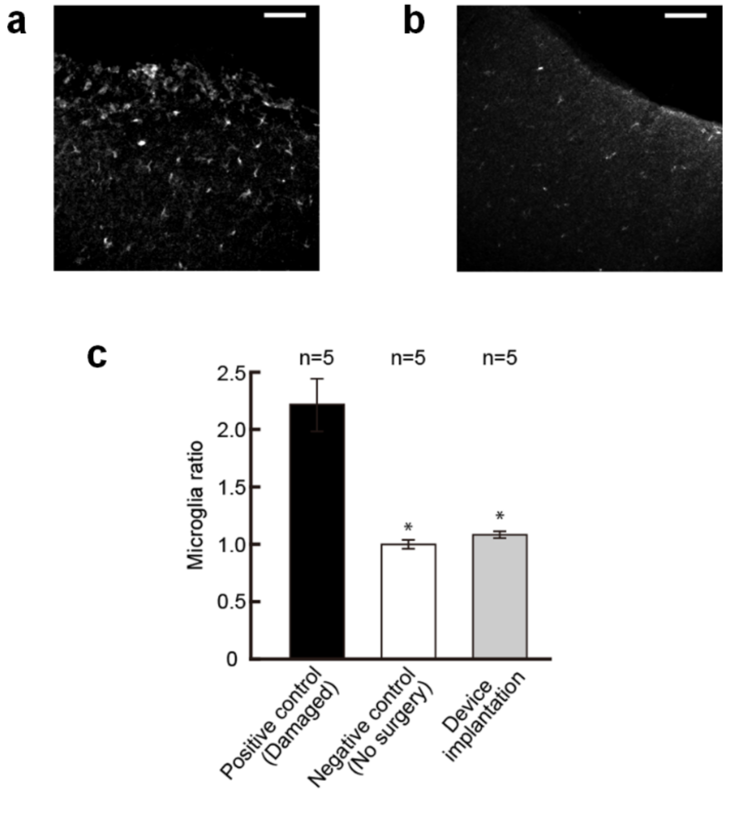


**Supplementary Figure S2** Assessment of physiological condition of the brain tissue by anti-Iba1 immunostaining of microglia. (a) Brain tissue after implantation procedure with mechanical stimulation (positive control). (b) Brain tissue after device implantation by low invasive device implantation procedure. (c) Expression level of microglial cells as a marker of the inflammatory reaction. Error bars, s.e.m.; *n*=5; positive control versus negative control and device implantation; * $P < 0.01$. Scale bars, 100 μm in a and b.

## References of supplementary information

1. Oheim, M., Beaurepaire, E., Chaigneau, E., Mertz, J. & Charpak, S. Two-photon microscopy in brain tissue: parameters influencing the imaging depth. *J. Neurosci. Meth.* **111**, 29-37 (2001).
2. Barton, K.A., Shui, Y.B., Petrash, J.M. & Beebe, D.C. Comment on: the Stokes–Einstein equation and the physiological effects of vitreous surgery. *Acta Ophthalmol.* **85**, 339-340 (2007).
3. Thorne, R.G. & Nicholson, C. In vivo diffusion analysis with quantum dots and dextrans predicts the width of brain extracellular space. *PNAS* **103**, 5567-5572 (2006).
4. Holtmaat, A. *et al.* Long-term, high-resolution imaging in the mouse neocortex through a chronic cranial window. *Nat. Protoc.* **4**, 1128-1144 (2009).
5. Xu, H.T., Pan, F., Yang, G. & Gan, W.B. Choice of cranial window type for in vivo imaging affects dendritic spine turnover in the cortex. *Nat. Neurosci.* **10**, 549-551 (2007).
